# Recommendation for ophthalmic care in German preschool health examination and its adherence: Results of the prospective cohort study ikidS

**DOI:** 10.1371/journal.pone.0208164

**Published:** 2018-12-03

**Authors:** Alexander K. Schuster, Heike M. Elflein, Christiane Diefenbach, Christine Gräf, Jochem König, Martina F. Schmidt, Kathleen Schnick-Vollmer, Michael S. Urschitz, on behalf of the ikidS-Study Group

**Affiliations:** 1 Department of Ophthalmology, University Medical Center of the Johannes Gutenberg-University, Mainz, Germany; 2 Division of Pediatric Epidemiology, Institute of Medical Biostatistics, Epidemiology and Informatics, University Medical Center of the Johannes Gutenberg-University, Mainz, Germany; University of Campania, ITALY

## Abstract

**Background:**

Each child in Germany undergoes a preschool health examination including vision screening and recommendations for further ophthalmic care. This study investigated the frequency of and adherence to these recommendations.

**Methods:**

A population-based prospective cohort study was performed in the area of Mainz-Bingen (Rhineland-Palatinate, Germany). All preschoolers were examined at the statutory preschool health examination, which includes vision testing (Rodenstock vision screener) with available correction in the last preschool year. Based on the results, recommendations for further ophthalmic care were given to the parents. Six weeks prior to school entry, parents were surveyed concerning ophthalmic health care visits, diagnoses, and treatments. Ophthalmic care recommendation frequency and its adherence were investigated using logistic regression analysis adjusted for potential confounders.

**Results:**

1226 children were included in this study, and 109 children received a recommendation for ophthalmic care based on the preschool health examination. At the follow-up, 84% of children who had received a recommendation had visited an ophthalmologist within the preceding year compared to 47% of children who had not received a recommendation. The recommendation for ophthalmic care was clearly associated with a higher number of ophthalmological visits (odds ratio = 7.63; 95% confidence interval: 3.96–14.7). In a subgroup analysis, adherence to a recommendation was lower in children with migrant background (OR = 2.26; 95%-CI: 0.64–7.90, compared to: OR = 11.6; 95%-CI: 4.95–27.4) and in those with low socio-economic status.

**Conclusions:**

Adherence to preschool recommendations for ophthalmic care is high in German preschoolers. However, a migrant background and low socio-economic status may reduce this adherence.

## Background

Adequate vision is not only related to quality of life, but also required for success at school [1]. The most common reason for reduced vision in children is uncorrected refractive error, while amblyopia caused by strabismus or anisometropia is the most common cause of uncorrectable vision loss in childhood and early adulthood [2, 3]. About 5% of children at school age suffer from strabismus [4] and between 2 and 7% have a myopic refractive error that needs optical correction [5]. Adequate correction and regular check-ups are necessary to lower the risk of developing amblyopia. Especially when entering school, adequate distance vision is necessary in order to follow lessons, particularly for teacher-centered teaching. Therefore, vision problems should be detected and adequately treated prior to school entry.

One aim of the preschool health examination (PHE) is to detect and resolve health impairments relevant for academic achievement. The PHE is carried out by Federal Public Health Services in Germany and, as one of several examinations, vision is screened. The screening includes measurement of visual acuity, binocular vision, and color discrimination. Based on these findings, a recommendation is given as to whether the child should visit an ophthalmologist for further diagnostic evaluation.

To date, there has been no evaluation of this vision screening in Germany. Parent-adherence to the given recommendations would justify this public health service. We analyzed the frequency of screening-associated recommendations and adherence to these recommendations in a cohort of preschool children.

## Methods

### Subjects

The present study analyzed data from the ikidS study, an ongoing population-based, prospective, closed cohort study located within the city limits of Mainz and the rural district of Mainz-Bingen (Rhineland-Palatinate; Germany). Methodological aspects and comparisons for representativeness of the cohort have been reported elsewhere [6].

In brief, all 79 public and private primary and special needs schools within the study region were included in this study. The source population of the cohort were the 3683 children who: i) were officially registered for school entry in 2015 in one of the 79 regional schools, and ii) had their PHE between September 1^st^, 2014, and August 31^st^, 2015. For the current analysis, children who ultimately did not enter school in September 2015 or were lost to follow up were excluded.

The study was approved by the ethics committee of the Medical Chamber of Rhineland-Palatinate, Germany, the regional supervisory school authority, and the state representative for data protection in Rhineland-Palatinate. Informed written parental consent was obtained.

### Baseline assessments (T0)

Data were collected at two time points: 1) at the PHE during the last preschool year (baseline, T0) and 2) six weeks before school entry, which corresponds to the beginning of the summer holidays (follow-up, T1).

The PHE is a standardized, compulsory, state-wide health examination performed by public youth health physicians employed by the regional Department of Public Health (Mainz-Bingen District). The parental PHE questionnaire gathers information on the age and sex of the child, parental education level, spoken language at home, whether the child requires glasses, and whether an ophthalmologist has been consulted within the preceding 12 months. At the PHE, visual acuity was tested wearing walk-in correction. A screening test was conducted using a Rodenstock Vision Tester (R11 or R21; sign: Test slice 120: tumbling E). Monocular visual acuity was tested for distance vision with habitual correction presenting letters for 20/30 (in decimal 0.7, logMAR = 0.18) and for 20/20 (in decimal 1.0, logMAR = 0.00). In addition, vision was tested with additional correction of +1.5D to identify hyperopia. Stereoscopic vision (Lang-I resp. Lang-II test or DeKa-test) and color discrimination (Ishihara test tables 11 and 14 for red-green deficiency) were also tested.

Based on these findings, a recommendation to visit an ophthalmologist for further diagnostic evaluation was given when monocular visual acuity was <0.7 in at least one eye in children without glasses or when visual acuity with additional correction of +1.5D improved. In children with glasses, the recommendation was only given when the last ophthalmic visit was more than 6 months ago. All children wearing glasses whose last ophthalmic visit was more than 12 months in the past also received the recommendation. Children who squinted in the general examination or who had misalignment in the cover test or did not recognize all figures on stereoscopic tests were also referred to an ophthalmologist. The recommendation was explained to the parents on the day of the PHE, and a letter to the child’s pediatrician and the parents was sent within 2 weeks after the PHE.

### Follow-up assessments (T1)

Six weeks prior to school entry (T1), the child’s general and mental health, the presence of chronic health conditions, any need for and use of special health care (including the consultation of an ophthalmologist), family structure and burden, leisure time activities, nutritional habits, environmental conditions, and socio-economic status were assessed by using a study-specific parental questionnaire.

A child was defined as having a migrant background if the child and one parent were not born in Germany, or when both parents were not born in Germany and/or did not have German citizenship. The presence of a chronic health condition was evaluated by a German version of the Children with Special Health Care Needs (CSHCN) Screener [7]. The CSHCN Screener is a 14-item parent-reported instrument which covers five different aspects of medical and psycho-social care, including medication, the need for social or educational support, need for physical, occupational, or speech therapy, having functional limitations, and mental problems requiring interventions. The screener indicates a chronic health condition if at least one of the five aspects is positively answered.

If there was any suspicion of visual dysfunction at the PHE, parents received an additional questionnaire focusing on recent ophthalmic care and any diagnoses received. Items covered the type of visual impairment (myopia, hyperopia, astigmatism, strabismus, other), the recommendations given at the PHE, and the type and extend of ophthalmic care since the PHE (S1 File and S2 File).

### Statistical analysis

Descriptive statistics such as absolute and relative frequencies, means, and standard errors were used for demographic and clinical characteristics. The frequency of recommendation to visit an ophthalmologist was computed for the study sample and stratified by age at the PHE, by gender, by socio-economic status, by migrant background, by the need to wear glasses, and by the presence of a chronic health condition; the frequency of adherence to this recommendation was calculated using the same stratifications. “Adherence” was defined as having received the recommendation at T0 and reporting to have visited an ophthalmologist within the 12 months prior to school entry (T1).

Unadjusted and adjusted effect estimates, p-values, and 95% confidential intervals were calculated using binary logistic regression analysis. Effect estimates (odds ratios) were adjusted for the following potential confounders: age, gender, socio-economic status, migrant background, wearing glasses, and the presence of a chronic health condition.

To evaluate effect modification, analyses were stratified by gender, migrant background, and socio-economic status. Socio-economic status was assessed analogously to a large German health survey [8] namely as a continuous variable and then later categorized into low (≤11.3), medium (>11.3 and <15.9) and high status (≥15.9). Analyses were carried out using the IBM Statistical Package for the Social Sciences (SPSS) version 24.0.

## Results

### Subjects

**Fig 1:**
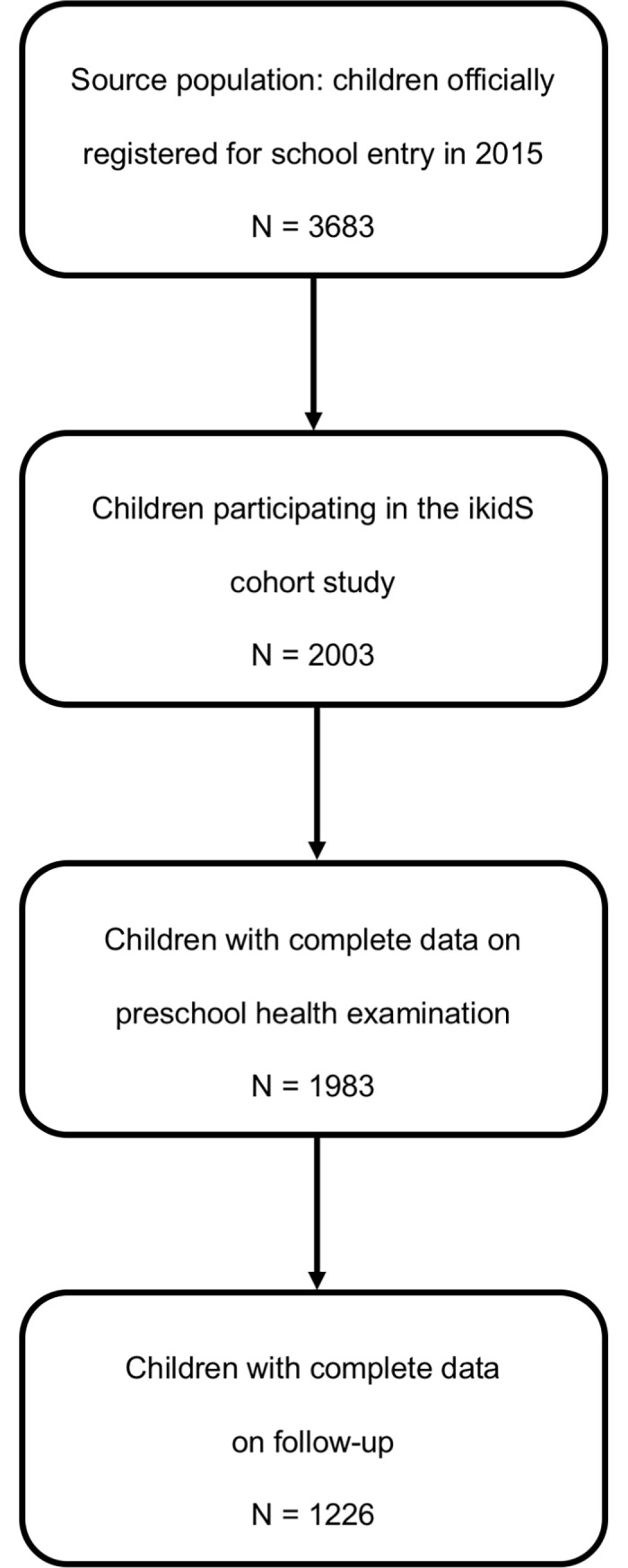
Flow chart of study participation.

Of 3683 eligible children, 2003 (i.e. 54% of the study population) were enrolled into the cohort. Cohort participants were fully representative of all children in the study region officially registered for school entry in 2015 [6]. Of these, 777 children were excluded for the present study, largely due to missing data concerning ophthalmic care at follow-up. The resulting study sample of 1226 children (Fig 1) was comparable to the underlying study population, except for migrant background (fewer children with A migrant background could be included in the present analysis; Table 1).

**Table 1:** Characteristics of eligible children and children enrolled into the study (i.e. participants with complete data from the preschool health examination and the follow-up survey immediately before school entry).

Characteristic	Underlying study population N = 3683	Study sample N = 1226
Sex (female)	48.1% (1774)	48.9% (599)
Age (at PHE): [mean +/- SD]	5.86 +/- 0.42	5.87 +/- 0.37
Migrant background (yes)	22.3% (822, NA = 464)	14.4% (177, NA = 58)
Socio-economic status:	Not available	13.8 +/- 4.0 (NA = 126)
Chronic diseases (CSHCN) (yes)	Not available	15.6% (191, NA = 25)
Visiting an ophthalmologist in the year before PHE (yes)	28.7% (1056, NA = 23)	36.1% (443)
Recommendation for ophthalmic care (yes)	10.8% (396)	8.9% (109)
Wearing glasses (yes)	6.7% (246)	7.7% (94)
Visual acuity (distance; in decimal) (right eye):		
<20/30 (decimal 0.7)	7.2% (265, NA = 307)	6.4% (78, NA = 97)
20/30 - <20/20 (decimal 0.7 - <1.0)	4.8% (176, NA = 396)	6.0% (73, NA = 102)
Visual acuity (distance; in decimal) (left eye):		
<20/30 (decimal 0.7)	7.4% (271, NA = 299)	6.8% (83, NA = 80)
20/30 - <20/20 (decimal 0.7 - <1.0)	4.6% (170, NA = 400)	4.6% (56, NA = 107)
Absence of stereopsis (yes)	4.2% (154, NA = 50)	3.6% (44, NA = 9)
Red-green deficiency (yes)	2.1% (78, NA = 42)	2.2% (27, NA = 7)

Unless otherwise stated, characteristics are summarized by total numbers (N) and frequencies (%). Abbreviations: PHE, preschool health examination.

In total, 109 children (8.9% of the study sample) received a PHE recommendation for further ophthalmic care; the reasons for the recommendations are described in S1 Table. 79 of these children had a visual acuity of <20/30 (<0.7) in at least one eye and 16 did not pass the stereoscopic test (Table 2). In some children these abnormalities were already known and the reasons for further ophthalmic care had been determined outside the actual PHE recommendation. In comparison, 97% of children without a recommendation had a visual acuity ≥20/30 (≥ 0.7) in each eye, 97% passed the stereoscopic test, and 99% did not show a red-green deficiency.

**Table 2:** Ophthalmic characteristics of the study sample, stratified by recommendation for ophthalmic care (N = 1,226).

Characteristic	No recommendation for ophthalmic care N = 1117	Recommendation for ophthalmic care N = 109
Visual acuity (right eye): <0.7 (distance)	20 (2%), NA = 83	58 (53%), NA = 14
Visual acuity (left eye): <0.7 (distance)	22 (2%), NA = 67	61 (56%), NA = 13
Visual acuity (in any eye): <0.7 (distance)	29 (3%), NA = 90	79 (72%), NA = 9
Absence of stereopsis (yes)	28 (3%), NA = 9	16 (15%)
Red-green deficiency (yes)	14 (1%), NA = 7	13 (12%)
Wearing glasses (yes)	87 (8%)	7 (6%)
Visiting an ophthalmologist in the year before school entry healthy examination (yes)	409 (37%)	34 (32%)

Unless otherwise stated, characteristics are summarized by total numbers (N) and frequencies (%). Abbreviations: NA, number of missing data.

Of the 109 children with a PHE recommendation for further ophthalmic care, 91 parents (83.5%) reported at follow-up that they had visited an ophthalmologist within the preceding year. Of the 1117 children without a PHE recommendation for ophthalmic care, 529 (47.3%) had visited an ophthalmologist within the year before (S2 Table).

Analyses limited to the children without any ophthalmic care before the PHE (N = 783) showed that 77% (58 of 75) of children having received a PHE recommendation visited an ophthalmologist, compared to 24% (168 of 708) of children without a PHE recommendation.

In regression analysis, the PHE recommendation for ophthalmic care was associated with higher odds of ophthalmological visits (unadjusted analysis: OR = 5.62; 95%-confidence interval [95%-CI]: 3.34–9.44; adjusted analysis: OR = 7.63; 95%-CI: 3.96–14.7; Table 3).

**Table 3:** Associations between PHE recommendation and other baseline characteristics with ophthalmic care (N = 1226).

Factors related to ophthalmic care prior to school entry	N	Have visited an ophthalmologist N (%)	Unadjusted OR [95%-CI]	Adjusted OR [95%-CI]
**PHE recommendation for ophthalmic care**				
Yes	109	91 (84%)	5.62 [3.34–9.44]	7.62 [3.96–14.7]
No	1117	529 (47%)	Reference	Reference
**Wearing spectacles**				
Yes	94	89 (95%)	20.1 [8.12–50.0]	27.7 [8.61–88.9]
No	1132	531 (47%)	Reference	Reference
**Age at PHE (per year)**	1226	Not applicable	0.90 [0.66–1.22]	1.00 [0.70–1.44]
**Gender**				
Female	599	320 (53%)	1.25 [1.00–1.57]	1.33 [1.02–1.73]
Male	627	300 (48%)	Reference	Reference
**Socio-economic status**				
Low	317	162 (51%)	0.97 [0.72–1.30]	0.97 [0.69–1.36]
Medium	367	185 (50%)	0.94 [0.71–1.25]	0.98 [0.72–1.33]
High	416	216 (52%)	Reference	Reference
**Migrant background**				
Yes	177	75 (42%)	0.67 [0.49–0.93]	0.68 [0.44–1.03]
No	991	518 (52%)	Reference	Reference
**Presence of a chronic health condition**				
Yes	191	109 (57%)	1.37 [1.00–1.87]	1.25 [0.87–1.82]
No	1010	497 (49%)	Reference	Reference

Results are given as unadjusted and adjusted odds ratios with 95% confidence intervals as calculated by binary logistic regression analysis.

Stratification by gender did not reveal relevant differences (adjusted analysis, boys: OR = 7.95; 95%-CI: 2.99–21.1; adjusted analysis, girls: OR = 7.55; 95%-CI: 3.09–18.4). A trend of effect modification was found with respect to socio-economic status (adjusted analysis, low status: OR = 4.00; 95%-CI: 1.62–9.83; adjusted analysis, medium status: OR = 9.00; 95%-CI: 2.62–31.0; adjusted analysis, high status: OR = 25.2; 95%-CI: 3.37–188; S3 Table). For the latter regression analysis, wearing glasses was excluded from the analysis due to model instability.

With respect to migrant background, the PHE recommendation for ophthalmic care led to higher odds of ophthalmic visits in children without a migrant background (adjusted analysis: OR = 11.6; 95%-CI: 4.95–27.4) compared to children with a migrant background (adjusted analysis: OR = 2.26; 95%-CI: 0.64–7.90; S4 Table).

Data from the additional study questionnaires focusing on ophthalmic care were available for 75 (69%) of 109 children: 26 (35%) of 75 parents reported that they had been aware of the ophthalmic pathology prior to the PHE, while 49 (65%) of 75 parents had not been aware of any eye disease prior to the PHE. Of the latter, 33 (67%) children visited an ophthalmologist after the PHE, as recommended. Of these, an ophthalmic pathology was ruled out in 21 children (66%), one (3%) child received further examinations, and an ophthalmic abnormality was confirmed in 10 (31%) children (parents of one child did not answer this question). Of the latter 10, seven parents reported a newly detected refractive error (three reported myopia, one having additional astigmatism; three reported hyperopia, one with additional astigmatism; one solely reported astigmatism), one parent reported strabismus, and two parents reported color deficiency.

## Discussion

Various countries have introduced PHEs to identify and treat health-related learning barriers including sensory deficits such as vision and hearing impairments, mental and behavioral problems, dental pain, and physical restrictions such as persistent hunger, obesity, and uncontrolled asthma [9, 10]. Most of these barriers have been identified as being linked with low school performance and reduced speech-language development [11–18].

Visual tasks are of large importance for learning success: most of the learning at school occurs through reading, writing, and using computers. Vision problems are linked to lower reading performance [19], and solving vision problems might result in improved school performance. Nevertheless, the impact of a vision screening at PHE is not well studied, and whether proposed recommendations are transferred into action remains unclear. In the present study, 84% of children having received a recommendation subsequently visited an ophthalmologist prior to school entry compared to 47% of children not having received such a recommendation. Thus, the recommendation for ophthalmic care at PHE was clearly associated with an increased use of ophthalmologic health care services. In a stratified analysis, adherence to the recommendation was lower in children with a migrant background and in those with low socio-economic status.

Prevalence estimates of vision impairments vary across different populations and with respect to specific vision issues: while some studies from the US report up to 22–30% of vision screen-positives [18, 20–22], the prevalence in our study sample was considerably low (i.e. 9%). This lower prevalence might be due to different screening instruments and procedures across different countries. In Germany, regular health examinations including functional visual tasks are solely carried out by pediatricians to detect children with vision problems. In other countries, screening is carried out by pediatricians, school health nurses, and/or other care deliverers [23–26].

Compliance to follow the recommendation for further ophthalmic examinations was generally high in our study: 84% stated to have visited an ophthalmologist in the year before entering school when receiving this recommendation. Despite this, we found that low socio-economic status of the family and a migrant background of the child were related to impaired adherence. This “social gradient” is well known from health services research and affects the uptake of childhood screening programs, the receipt of pediatric healthcare in general, and specialist care such as ophthalmic care in particular [27]. This is of importance as low socio-economic status and having a migrant background are *per se* well known risk factors of poor school performance [28–30]. Thus, identification and treatment of health-related learning barriers in these at-risk children are major public health goals.

Most of the ophthalmic abnormalities detected were refractive errors that can be corrected with glasses, while one case of strabismus was identified. While the last preschool year allows sufficient detection of refractive error, it is general accepted that screening of even younger children and subsequently early treatment of amblyopia may result in better visual acuity.

Finally, out study has some limitations. First, we were not able to validate the parental reports of having visited an ophthalmologist in the last year. Second, the time frame for having visited an ophthalmologist was not clearly limited to the time period since the PHE, but generalized to the last year before answering the questionnaire. This enabled us to compare healthcare uptake in the cohort at different grades in school, but was not targeted to the present research questions. Third, we did not have access to the records of the ophthalmic examination of the children with a positive screening, but rather data obtained by a questionnaire with respect to ophthalmic care since PHE. The PHE vision screening was based on visual acuity measures, which may have low sensitivity and specificity for detection of hyperopia and astigmatism [31]. Therefore, some children with hyperopia and astigmatism may have been missed. Due to this limitation, vision screening with additional correction of +1.5D was added to the screening procedure. Using tumbling E as the target in the German PHE vision screening requires discrimination of rotation and sufficient verbal skills by the respective child. This may not be sufficiently developed in preschool children [32]. In contrast, HOTV letters and LEA symbols may not show this limitation [33]. Nevertheless, a recent study showed high feasibility rates in European Caucasian children at age 3–4 years [34]. In the past, the diagnostic accuracy of the Rodenstock device has been investigated using pictures instead of tumbling the E target and showed a positive predictive value of 67% and negative predictive value of 80% [35].

In summary, most parents follow the recommendation for further ophthalmic examination when there is a positive vision screening test result at the PHE. Children living in low socio-economic conditions and having a migrant background are at risk of not receiving further ophthalmic care, which may impair their educational progress.

## Supporting information

S1 Table Reasons of recommendations for further ophthalmic care (N = 109). (DOCX)

S2 Table Cross-tabulation between recommendation for ophthalmic care and adherence to this recommendation (N = 1,226). (DOCX)

S3 Table Cross-tabulation between recommendation for ophthalmic care and adherence to this recommendation, stratified by socio-economic status (N = 1,100). (DOCX)

S4 Table Cross-tabulation between recommendation for ophthalmic care and adherence to this recommendation, stratified by migrant background (N = 1,168). (DOCX)

S1 Dataset Legend for dataset of the prospective cohort study ikidS for evaluation of recommendation for ophthalmic care in German preschool health examination and its adherence. (XLSX)

S2 Dataset Dataset of the prospective cohort study ikidS for evaluation of recommendation for ophthalmic care in German preschool health examination and its adherence. (CSV)

S1 File Translated questionnaire “vision problems” of the prospective cohort study ikidS. (DOCX)

S2 File German questionnaire “vision problems” of the prospective cohort study ikidS. (DOCX)
