# Supplementary material for: Recommendation for ophthalmic care in German preschool health examination and its adherence: Results of the prospective cohort study ikidS
Source: PLoS One. 2018 Dec 3;13(12):e0208164. doi: 10.1371/journal.pone.0208164 (PMC6277132; doi:10.1371/journal.pone.0208164)
Supplement: S1 Table — (DOCX) [file pone.0208164.s001.docx]

**S1 Table. Reasons of recommendations for further ophthalmic care (N = 109).**

| **Reason** | **N** | **%** |
| --- | --- | --- |
| Stereoscopic test showing abnormality | 15 | 14% |
| Visual acuity <0.7 in at least one eye without glasses | 73 (thereof 8 with recommendation due to stereoscopic test) | 67% |
| Visual acuity <0.7 in at least one eye with glasses and no ophthalmological visit within the last year | 2 | 2% |
| Other difficulties/ abnormalities when performing the examination | 27 | 25% |
