# Supplementary material for: Recommendation for ophthalmic care in German preschool health examination and its adherence: Results of the prospective cohort study ikidS
Source: PLoS One. 2018 Dec 3;13(12):e0208164. doi: 10.1371/journal.pone.0208164 (PMC6277132; doi:10.1371/journal.pone.0208164)
Supplement: S2 Table — (DOCX) [file pone.0208164.s002.docx]

**S2 Table. Cross-tabulation between recommendation for ophthalmic care and adherence to this recommendation (N = 1,226).**

|  | | Having visited an ophthalmologist prior to school entry | |
| --- | --- | --- | --- |
|  |  | **No (n=606)** | **Yes (n=620)** |
| **PHE recommendation to visit an ophthalmologist** | **No (n=1117)** | 588 (53%) | 529 (47%) |
|  | **Yes (n=109)** | 18 (16%) | 91 (84%) |
