# Supplementary material for: Recommendation for ophthalmic care in German preschool health examination and its adherence: Results of the prospective cohort study ikidS
Source: PLoS One. 2018 Dec 3;13(12):e0208164. doi: 10.1371/journal.pone.0208164 (PMC6277132; doi:10.1371/journal.pone.0208164)
Supplement: S3 Table — (DOCX) [file pone.0208164.s003.docx]

**S3 Table. Cross-tabulation between recommendation for ophthalmic care and adherence to this recommendation, stratified by socio-economic status (N = 1,100).**

| **Socioeconomic status** | **PHE recommendation to visit an ophthalmologist** | | Having visited an ophthalmologist prior to school entry | |
| --- | --- | --- | --- | --- |
|  |  |  | **No (n=155)** | **Yes (n=162)** |
| **Low** (N=317) | **PHE recommendation to visit an ophthalmologist** | **No (n=284)** | 148 (52%) | 136 (48%) |
|  |  | **Yes (n=33)** | 7 (21%) | 26 (79%) |
|  | | | | |
|  |  |  | **No (n=182)** | **Yes (n=185)** |
| **Medium** (N=367) | **PHE recommendation to visit an ophthalmologist** | **No (n=339)** | 179 (53%) | 160 (47%) |
|  |  | **Yes (n=28)** | 3 (11%) | 25 (89%) |
|  | | | | |
|  |  |  | **No (n=200)** | **Yes (n=216)** |
| **High** (N=416) | **PHE recommendation to visit an ophthalmologist** | **No (n=390)** | 199 (51%) | 191 (49%) |
|  |  | **Yes (n=26)** | 1 (4%) | 25 (96%) |
|  | | | | |
|  |  |  | **No (n=69)** | **Yes (n=57)** |
| **Missing** (N=126) | **PHE recommendation to visit an ophthalmologist** | **No (n=104)** | 62 (60%) | 42 (40%) |
|  |  | **Yes (n=22)** | 7 (32%) | 15 (68%) |
