# Supplementary material for: Recommendation for ophthalmic care in German preschool health examination and its adherence: Results of the prospective cohort study ikidS
Source: PLoS One. 2018 Dec 3;13(12):e0208164. doi: 10.1371/journal.pone.0208164 (PMC6277132; doi:10.1371/journal.pone.0208164)
Supplement: S4 Table — (DOCX) [file pone.0208164.s004.docx]

**S4 Table. Cross-tabulation between recommendation for ophthalmic care and adherence to this recommendation, stratified by migrant background (N = 1,168).**

| **Migrant background** | **PHE recommendation to visit an ophthalmologist** | | Having visited an ophthalmologist prior to school entry | |
| --- | --- | --- | --- | --- |
|  |  |  | **No (n=473)** | **Yes (n=518)** |
| **No** (N=991) | **PHE recommendation to visit an ophthalmologist** | **No (n=906)** | 462 (51%) | 444 (49%) |
|  |  | **Yes (n=85)** | 11 (13%) | 74 (87%) |
|  | | | | |
|  |  |  | **No (n=102)** | **Yes (n=75)** |
| **Yes** (N=177) | **PHE recommendation to visit an ophthalmologist** | **No (n=155)** | 95 (61%) | 60 (39%) |
|  |  | **Yes (n=22)** | 7 (32%) | 15 (68%) |
|  | | | | |
|  |  |  | **No (n=31)** | **Yes (n=27)** |
| **Missing** (N=58) | **PHE recommendation to visit an ophthalmologist** | **No (n=56)** | 31 (55%) | 25 (45%) |
|  |  | **Yes (n=2)** | 0 (0%) | 2 (100%) |
