# Supplementary material for: Recommendation for ophthalmic care in German preschool health examination and its adherence: Results of the prospective cohort study ikidS
Source: PLoS One. 2018 Dec 3;13(12):e0208164. doi: 10.1371/journal.pone.0208164 (PMC6277132; doi:10.1371/journal.pone.0208164)
Supplement: S1 File — (DOCX) [file pone.0208164.s007.docx]

Dear Parents,

At the preschool health examination, you stated that your child has a vision problem/refractive error or the school physician suspected a vision problem. With the following questionnaire, we further examine the seriousness of this vision problem and the related effects on your child and your family.

**Questions concerning vision problems and refractive error**

**1a. Had there been hints as to a vision problem before the preschool health examination or had there been a diagnosis of vision problems?**

No ...

Yes... **⇨** **If Yes, go to question 2**

1b. Had there been a hint as to a vision problem during the preschool health examination (i.e. at the vision test)?

No ... **⇨** **If No, the questionnaire on vision problems ends here.**

Yes...

**1c. Did you go to see an ophthalmologist to clarify the suspicion of a vision problem after the preschool health examination?**

No ... **⇨** **If No, the questionnaire on vision problems ends here.**

Yes...

**1d. Was the vision problem confirmed by an ophthalmologist?**

**Yes,** an ophthalmologist confirmed the vision problem ...

**No,** an ophthalmologist ruled out a vision problem ...

**No,** not yet, the ophthalmologist wants to perform further tests...

**⇨** **If No, the questionnaire on vision problems ends here.**

**2. Which vision problems or refractive error does your child have?** *(Several answers are possible)*

Myopia …□ Hyperopia...□

Astigmatism...□ Strabismus.…□

Other eye diseases...□ Please describe: ________________________

Don’t know ...□

**3. At what age was a vision problem of your child confirmed?**

At _____years ______ month Don’t know ...□

**4. Who confirmed the vision problem of your child and/or treated it?** *(Several answers are possible)*

Confirmed Treated

General practitioner …□ …□

Pediatrician …□ …□

Physician at school entry examination. …□ …□

Optician / Optometrist □ …□

Ophthalmologist in practice …□ …□

Eye clinic …□ …□

Special out-patient eye clinic □ …□

others… □ …□,

If others, please describe the specialty: _______________ _________

**5. Do you regularly go to ophthalmic controls with your child?**

No …□ Yes …□, every _____ months.

**6. Have you ever occluded an eye of your child due to vision problems?**

No ...□ **⇨** **If No, please go to question 8.**

Yes ...□

**7. Have you occluded an eye of your child within the last 12 months?**

No ...□ Yes ...□

If Yes, for how many hours per day did occluded one eye? For ____hours

If Yes, does it still have to be occluded? No ...□ Yes ...□

**8. Did your child receive a prescription for glasses/contact lenses?**

No …□ **⇨** **If No, please go to question 12.**

Yes ...□

**9. At what age did your child receive glasses/contact lenses?**

At _____years ______ month Don’t know ...□

**10. When does your child wear his glasses/contact lenses?** *(Several answers are possible)*

The whole day...□ at school...□ while doing homework...□

**11. Does your child use glasses/contact lenses as prescribed by the ophthalmologist?**

Always...□ Often...□ Barely to never...□

**12. Does your child need vision aids (e.g. a magnifying glass) or other aids to improve vision?**

No ...□ Yes ...□

**13. Does your child have particular problems at school due to vision problems?**

No... □ Yes... □

If Yes, please describe: _______________________________________________________________________ _____________________________________________________________________________________________________________________________________________________________________________________________________________________

**14. What could be done from your point of view to solve these problems?**

____________________________________________________________________________________________________________________________________________________________________________________________________________________________________________________________________________________________

**Thank you very much for your help!**
