# Supplementary material for: Recommendation for ophthalmic care in German preschool health examination and its adherence: Results of the prospective cohort study ikidS
Source: PLoS One. 2018 Dec 3;13(12):e0208164. doi: 10.1371/journal.pone.0208164 (PMC6277132; doi:10.1371/journal.pone.0208164)
Supplement: S2 File — (DOCX) [file pone.0208164.s008.docx]

Liebe Eltern,

im Rahmen der Schuleingangsuntersuchung hatten Sie angegeben, dass bei Ihrem Kind eine Sehstörung / Fehlsichtigkeit besteht oder es wurde von der Schulärztin beim Sehtest ein auffälliger Befund erhoben. Mit den folgenden Fragen erfassen wir das Ausmaß der Sehstörung und die damit verbundenen Belastungen für Ihr Kind und Ihre Familie.

**Fragen zu Sehstörungen und Fehlsichtigkeit**

**1a. Bestanden bereits vor der Schuleingangsuntersuchung Hinweise auf eine Sehstörung** **oder lag die Diagnose einer Sehstörung vor?**

Nein ...

Ja... **⇨** **Falls Ja, weiter mit Frage 2**

1b. Ergaben sich im Rahmen der Schuleingangsuntersuchung (z.B. durch den Sehtest) Hinweise auf eine Sehstörung?

Nein ... **⇨** **Falls Nein, ist an dieser Stelle die Befragung zur
 Sehstörung für Sie beendet.**

Ja...

**1c. Waren Sie nach der Schuleingangsuntersuchung bei einem Augenarzt um den Verdacht auf eine Sehstörung abzuklären?**

Nein ... **⇨** **Falls Nein, ist an dieser Stelle die Befragung zur
 Sehstörung für Sie beendet.**

Ja...

**1d. Wurde die Sehstörung durch den Augenarzt bestätigt?**

**Ja,** der Augenarzt hat die Sehstörung bestätigt ...

**Nein,** der Augenarzt hat die Sehstörung ausgeschlossen ...

**Nein,** bislang nicht, der Augenarzt möchte weitere Untersuchungen durchführen ...

**⇨** **Falls Nein, ist an dieser Stelle die Befragung zur Sehstörung für Sie beendet.**

**2. Welche Sehstörung oder Fehlsichtigkeit hat Ihr Kind?** *(Mehrfachantworten möglich)*

Kurzsichtigkeit …□ Weitsichtigkeit...□ Hornhautverkrümmung...□ Schielen .… □

Andere Augenerkrankung...□ Welche? ________________________

Weiß nicht ...□

**3. In welchem Alter wurde die Sehstörung Ihres Kindes festgestellt?**

Mit _____Jahren ______ Monaten Weiß nicht ...□

**4. Wer hat die Sehstörung Ihres Kindes bestätigt und/oder behandelt?** *(Mehrfachantworten möglich)*

Bestätigt Behandelt

Hausarzt …□ …□

Kinderarzt …□ …□

Schularzt bei Einschulungsu. …□ …□

Optiker / Optometrist □ …□

Niedergelassener Augenarzt …□ …□

Augenklinik …□ …□

Spezialambulanz einer Augenklinik ? □ …□

Andere… □ …□,

Falls andere, geben Sie bitte die Fachrichtung an_______________ _________

**5. Gehen Sie mit Ihrem Kind regelmäßig zu augenärztlichen Kontrolluntersuchungen?**

Nein …□ Ja …□, im Abstand von ungefähr _____ Monaten.

**6. Haben Sie Ihrem Kind wegen Sehschwäche jemals ein Auge abkleben müssen?**

Nein ...□ **⇨** **Falls nein, bitte weiter mit Frage 8.**

Ja ...□

**7. Haben Sie Ihrem Kind auch noch in den letzten 12 Monaten ein Auge abgeklebt?**

Nein ...□ Ja ...□

Falls ja, wie viele Stunden pro Tag haben Sie ein Auge abgeklebt? ____Stunden

Falls ja, muss aktuell noch immer abgeklebt werden? Nein ...□ Ja ...□

**8. Wurde Ihrem Kind eine Brille oder Kontaktlinsen verordnet?**

Nein …□ **⇨** **Falls nein, bitte weiter mit Frage 12.**

Ja ...□

**9. In welchem Alter hat Ihr Kind die Brille bzw. Kontaktlinsen bekommen?**

Mit _____Jahren ______ Monaten Weiß nicht ...□

**10. Wann trägt Ihr Kind seine Brille bzw. die Kontaktlinsen?** *(Mehrfachantworten möglich)*

Den ganzen Tag...□ In der Schule...□ Bei den Hausaufgaben...□

**11. Trägt Ihr Kind seine Brille bzw. die Kontaktlinsen wie vom Augenarzt verordnet?**

Immer...□ Oft...□ Selten bis nie...□

**12. Benötigt Ihr Kind vergrößernde Sehhilfen (z.B. Lupen) oder andere Hilfsmittel zur
 Verbesserung des Sehens?**

Nein ...□ Ja ...□

**13. Hat Ihr Kind durch die Sehstörung besondere Probleme in der Schule?**

Nein... □ Ja... □

Falls ja, welche? _______________________________________________________________________ _____________________________________________________________________________________________________________________________________________________________________________________________________________________

**14. Was könnte aus Ihrer Sicht getan werden, um diese Probleme zu beheben?**

____________________________________________________________________________________________________________________________________________________________________________________________________________________________________________________________________________________________

**Herzlichen Dank für Ihre Unterstützung!**
